# Encapsulation of Antifungals in Micelles Protects *Candida albicans* during Gall-Bladder Infection

**DOI:** 10.3389/fmicb.2017.00117

**Published:** 2017-02-01

**Authors:** Shih-Hung Hsieh, Sascha Brunke, Matthias Brock

**Affiliations:** ^1^Microbial Biochemistry and Physiology, Leibniz Institute for Natural Product Research and Infection Biology, Hans Knoell InstituteJena, Germany; ^2^Fungal Genetics and Biology Group, School of Life Sciences, University of NottinghamNottingham, UK; ^3^Microbial Pathogenicity Mechanisms, Leibniz Institute for Natural Product Research and Infection Biology, Hans Knoell InstituteJena, Germany

**Keywords:** conjugated bile salts, caspofungin, farnesol, rhodamine 6G, critical micelle concentration

## Abstract

*Candida albicans* is a dimorphic fungus that colonizes human mucosal surfaces with the potential to cause life-threatening invasive candidiasis. Studies on systemic candidiasis in a murine infection model using *in vivo* real-time bioluminescence imaging revealed persistence of *C. albicans* in the gall bladder under antifungal therapy. Preliminary analyses showed that bile conferred resistance against a wide variety of antifungals enabling survival in this cryptic host niche. Here, bile and its components were studied for their ability to reduce antifungal efficacy in order to elucidate the underlying mechanism of protection. While unconjugated bile salts were toxic to *C. albicans*, taurine, or glycine conjugated bile salts were well tolerated and protective against caspofungin and amphotericin B when exceeding their critical micellar concentration. Microarray experiments indicated that upregulation of genes generally known to mediate antifungal protection is not involved in the protection process. In contrast, rhodamine 6G and crystal violet in- and efflux experiments indicated encapsulation of antifungals in micelles, thereby reducing their bioavailability. Furthermore, farnesol sensing was abolished in the presence of conjugated bile salts trapping *C. albicans* cells in the hyphal morphology. This suggests that bioavailability of amphiphilic and hydrophobic compounds is reduced in the presence of bile. In contrast, small and hydrophilic molecules, such as cycloheximide, flucytosine, or sodium azide kept their antifungal properties. We therefore conclude that treatment of gall bladder and bile duct infections is hampered by the ability of bile salts to encapsulate antifungals in micelles. As a consequence, treatment of gall bladder or bile duct infections should favor the use of small hydrophilic drugs that are not solubilised in micelles.

## Introduction

The dimorphic fungus *Candida albicans* is frequently found as a commensal on mucosal surfaces. In addition, it causes life-threatening systemic infections in patients with risk factors, such as diabetes, central line catheters, cancer, or organ transplantation (Akpan and Morgan, 2002; Cassone and Cauda, 2012; Gavalda et al., 2014; Teoh and Pavelka, 2016). To control systemic candidiasis, the echinocandin caspofungin, the polyene macrolide amphotericin B or azoles are commonly used (Gullo, 2009; Li et al., 2015; Matthaiou et al., 2015). To follow antifungal therapy efficacy in a systemic murine infection model an *in vivo* bioluminescence imaging system was recently developed that visualizes invasive disease in real-time and in temporal and spatial resolution (Jacobsen et al., 2014). Systemic infection revealed a rapid manifestation of disease in kidneys with bioluminescence signals correlating with fungal burden. A subsequent therapy approach with initial caspofungin treatment and fluconazole de-escalation showed rapid clearance of infection from kidneys, and mice appeared clinically inconspicuous after initiation of treatment. Unexpectedly, some mice from the therapy group developed bioluminescence signals from the gall bladder (Jacobsen et al., 2014) and living *C. albicans* cells were shed with the release of bile from the gall bladder, potentially leading to a re-colonization of the intestinal tract. Preliminary analyses indicated that bile decreases the sensitivity of *C. albicans* against a wide variety of commonly used antifungals, implying that the gall bladder forms a protective niche during antifungal therapy (Jacobsen et al., 2014).

*Candida albicans* infections of the liver and biliary system have been described as a major complication in liver transplant recipients (Romero and Razonable, 2011; Hernandez Mdel et al., 2015) and a prophylactic therapy with antifungals, mainly with fluconazole or echinocandins is recommended (Hernandez Mdel et al., 2015). However, while a case study on a liver transplant recipient suffering from *Candida* cholangitis recommended caspofungin for treatment of biliary infections (Goicoechea et al., 2004) the study showed that despite parenteral caspofungin therapy fluconazole sensitive *C. albicans* could still be isolated from bile samples (Goicoechea et al., 2004). Another clinical case report showed that conventional amphotericin B failed to control candidemia in the gall bladder (Jajoo et al., 2012) and in a clinical case series *Candida* bile duct infections were linked to treatment failure in some patients (Domagk et al., 2006). Additional studies indicate that treatment of fungal infections of the biliary system appears to present a more general problem. One study showed that antifungal therapy was ineffective in a biliary *Coccidioides immitis* infection (Sydorak et al., 2001). In another case report early hepatic artery thrombosis due to bacterial and *Aspergillus* infection occurred in a liver transplant recipient. Despite antibiotic and amphotericin B treatment the infection was not resolved and a revision of liver transplantation was required. It was speculated that the donor liver was the primary source of the infection (Jafarian et al., 2014). These studies indicate that the gall bladder may serve as a fungal infection reservoir not only in a murine model of candidiasis, but also in humans.

Due to the reduced susceptibility of *C. albicans* toward antifungals in the presence of bile and the described problems of antifungal therapy in biliary tract infections we investigated the bile-mediated protective effect in more detail.

## Materials and Methods

### Strains and General Culture Conditions

If not indicated otherwise, *C. albicans* strain SC5314 was used throughout all experiments. For studies on Tye7, strain SN152 *tye7*Δ/Δ and the corresponding wild-type strain SN152 were used (Noble and Johnson, 2005; Homann et al., 2009). Pre-cultures were grown over night at 30°C in 20 ml YPD medium (per liter: 10 g yeast extract, 20 g peptone, and 20 g glucose). Cells were harvested by centrifugation at 4000 ×*g*, washed twice in phosphate-buffered saline (PBS) and suspended in either YPD or MOPS-buffered RPMI 1640 (Sigma) with 2% glucose (per liter: 10.4 g RPMI 1640, 34.53 g MOPS, 20 g glucose; pH 6.8; subsequently defined as RPMI medium). Stock solution of caspofungin (5 mg/ml; Cancidas^®^, Merck, Germany), flucytosine (10 mg/ml), cycloheximide (20 mg/ml; Sigma C7698) or sodium azide (20 mg/ml) were prepared in PBS and filter sterilized. Amphotericin B stock solution was prepared at 4 mg/ml in DMSO. Fluconazole solution at 2 mg/ml was purchased from B. Braun, Germany. All drugs were diluted in the respective media that were used for sensitivity analyses. Controls were prepared according to the solvents used for stock solutions of the respective antibiotics.

### Preparation of Bile and Bile Salt Containing Media

Bile stock solution (12.5%) was prepared by dissolving crude porcine bile extract (Sigma, B8631) in RPMI or YPD. Insoluble components were removed by centrifugation at 12000 ×*g*, the supernatant filter sterilized and stored at 4°C in the dark. Sodium taurodeoxycholate hydrate (Sigma, T0875), taurocholic acid sodium salt hydrate (Sigma, T4409), sodium glycocholate hydrate (Sigma, G7132), and unconjugated bile salts (UBS) (Sigma 48305, 50% cholic acid sodium salt and 50% deoxycholic acid sodium salt) were dissolved at 100 mg/ml in either YPD or RPMI, filtered and used as stock solutions.

### Drug Resistance Analyses

Antifungal drug resistance or toxicity of bile salts was tested in broth dilution assays. Precultures of *C. albicans* yeasts were grown for 16 h in liquid YPD, harvested by 10 min centrifugation at 4000 ×*g* and washed twice in PBS. Dilutions of bile or bile salts with or without antifungals were prepared in either RPMI medium or YPD and transferred to 96-well plates. *C. albicans* cells were pre-diluted in the respective growth medium and added to a final concentration of 4 × 10^4^ yeasts in 200 μl medium. NaCl in YPD was used to test the effect of ionic strength. pH effects were tested in RPMI medium with MOPS buffer adjusted to different pH values. Resistance against 1 μg/ml caspofungin was evaluated in the presence of either 2.5 mg/ml sodium taurodeoxycholate or 10 mg/ml sodium taurocholate. All plates were sealed with transparent gas permeable moisture barrier seal (4titude) and incubated at 37°C for 20 h, with OD_600_ measurements every 15 min preceded by 5 s shaking. Analyses were performed in triplicates with at least one biological replication. Data were analyzed by Microsoft Excel, and either growth curves or end point determinations are shown.

### Pre-adaptation Analyses

*Candida albicans* cells were pre-incubated in YPD medium for 16 h at 30°C in the presence of 12.5% (w/v) bile or 25 mg/ml sodium taurocholate (TC). Cells were washed once in PBS and transferred at an OD_600_ of 0.1 into 96-well plates containing MOPS-buffered RPMI with 2% glucose medium and different concentrations of amphotericin B or caspofungin and growth curves were recorded.

### Microarray Analyses

YPD-grown cells were washed with PBS and transferred at OD_600_ of 0.1 to MOPS-buffered RPMI 2% glucose medium and grown for 4 h at 37°C on a rotary shaker at 180 rpm. Subsequently, parallel cultures were supplemented with either 25 mg/ml TC or left as controls. Cells were harvested by centrifugation at 0.5 and 2 h and directly subjected to RNA extraction as previously described (Ramachandra et al., 2014). Microarrays were performed with Cy5-CTP labeled cRNA (GE Healthcare) using RNA from an exponentially growing *C. albicans* YPD liquid culture as Cy3-labeled common reference. Hybridisation, scanning and data analysis were performed as described (Ramachandra et al., 2014). The data was LOWESS normalized and evaluated using the GeneSpring GX software package, version 12.1 (Agilent). GO-Term analysis based on genome annotations from the *Candida* Genome Database (Inglis et al., 2012) was performed with the same program. Gene transcription data was deposited at the ArrayExpress database^1^ under accession number E-MTAB-5277.

### Quantitative Real Time PCR Analyses

*C. albicans* yeast cells were incubated for 4 or 20 h at 37°C in MOPS-buffered RPMI 2% glucose medium in presence or absence of 10 mg/ml TC. Cells were harvested by centrifugation, directly frozen in liquid nitrogen and RNA was extracted as described above. cDNA was produced by RevertAid Reverse Transcriptase (Thermo Scientific) with anchored oligo(dT) primers. qRT-PCR was analyzed on a CFX384 Touch Real-Time PCR Detection System (BioRad, Munich, Germany) using EvaGreen 5 × qPCR (ROX) mix (Bio & Sell, Feucht, Germany). The *ACT1* and *EFB1* genes served as reference for normalization. Normalized transcript levels were expressed as fold expression = Δ2^(reference^ ^-target)^ based on expression levels in absence of TC. Oligonucleotide sequences are provided in **Table 1**.

**Table 1:** Oligonucleotides used in qRT-PCR experiments.

*ECE1*	Forward: ACAGCCATGACTTCTGTTGC
	Reverse: ATCTGGAACGCCATCTCTCT
*UME6*	Forward: TGGTAATGGCACTAACACCAA
	Reverse: TGTAATCCAAATTTAGCACAACC
*HWP1*	Forward: CCAGGTGCTTCTTCTTCTCC
	Reverse: TAATTGGCAGATGGTTGCAT
*HGC1*	Forward: CAATTCTTCAACGTCATTAGGG
	Reverse: GCATTTGTGGTGGTGGTATC
*EFB1*	Forward: GTCGACTTGTTCGGTTCTGA
	Reverse: TTCGATAGCTTTGACGTTGG
*ACT1*	Forward: ATGGACGGTGGTATGTTTTAG
	Reverse: GAACAATGGATGGACCAGATTCGTCG

### Farnesol Effects in Presence of Taurocholic Acid

A 200 mM ethanolic stock solution of *trans*, *trans* farnesol was diluted 1000-fold in MOPS-buffered RPMI 2% glucose medium in presence or absence of 10 mg/ml TC. Shake flask cultures (5 ml) were inoculated with *C. albicans* yeasts at an OD_600_ of 0.1 and incubated at 37°C and 180 rpm. Samples were removed at various time points and subjected to microscopy on a Zeiss AxioImager M1.

### Rhodamine 6G and Crystal Violet In- and Efflux Analyses

*Candida albicans* yeasts were incubated at 30°C in MOPS-buffered RPMI 2% glucose medium in the presence of 1 μM Rhodamine 6G (R6G) and various concentrations of bile or conjugated bile salts. Aliquots were harvested at different time points, washed twice with 50 mM HEPES buffer pH 7.0. R6G fluorescence was measured in a microplate reader (Fluostar Omega, BMG Labtech) at 545 nm excitation and 590 nm emission. Fluorescence was normalized against OD_600_. For R6G efflux analyses, *C. albicans* yeasts were loaded for 30 min at 30°C with 1 μM R6G. Yeast cells were collected by centrifugation and suspended in fresh media containing 1 μM R6G supplemented with either bile, sodium taurocholate or sodium taurodeoxycholate (all at final concentration of 5 mg/ml) or left without supplementation. At different time points cells were harvested and washed once in HEPES buffer. The efflux of R6G was determined from the relative decrease of fluorescence from OD_600_ normalized cell densities. For crystal violet (CV) absorption analyses yeasts were incubated for 30 min at 30°C in MOPS-buffered RPMI 2% glucose medium containing 2 or 4 μg/ml CV. As indicated media contained 5 mg/ml bile or conjugated bile salts and different concentrations (0–80 mg/ml) of NaCl. Uptake of CV was visualized by photographs of cell pellets. Absorption rate was quantified at 590 nm from supernatants after cell removal and compared to mock-inoculated medium. Relative CV absorption (%) was calculated from (A590_control_-A590_sample_)/A590_control_ × 100.

### Determination of CMC Values

Critical micelle concentration (CMC) was determined as previously described (Patist et al., 2000) based on the change of absorbance of eosin Y during formation of micelles. In brief, 19 μM of eosin Y were mixed with different concentrations of either bile or taurine conjugated bile salts and absorbance was measured at 542 nm in a 96-well plate using a microplate reader (Fluostar Omega, BMG Labtech). Absorbance was plotted against the log concentration of the solubilising agent for calculation of the CMC.

### Statistical Analyses

Results in bar diagrams are shown as mean ± standard deviation (SD). Growth curves depict mean values from technical and biological replicates. All experiments were performed in biological duplicates or triplicates with two or three wells inoculated in parallel. Statistical significance was calculated using the two-tailed Student’s *t*-test.

## Results

### Proteins, Phospholipids, and Cholesterol Do Not Protect against Antifungals

**FIGURE 1:**
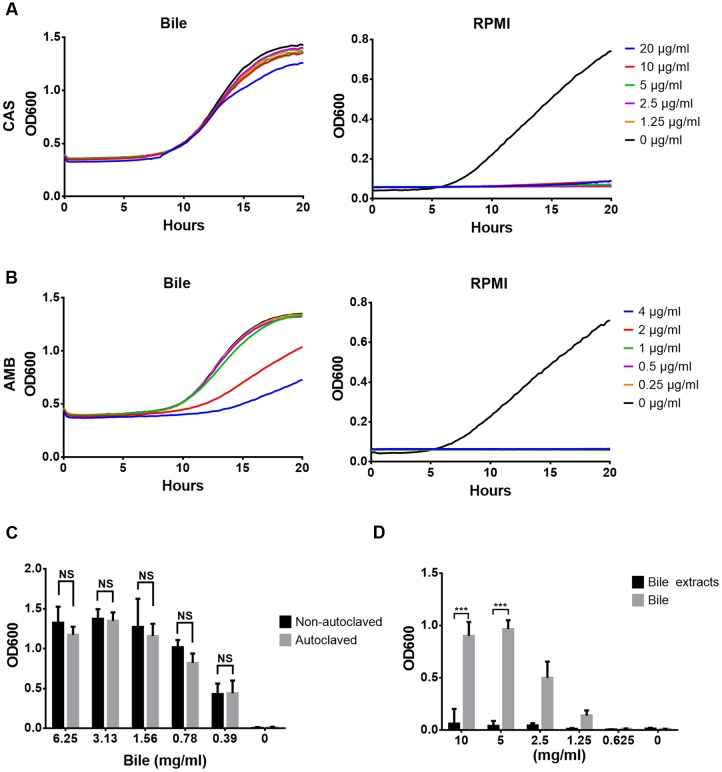
**Antifungal resistance against caspofungin and amphotericin B in presence of bile and bile extracts.** Experiments were performed in biological and technical triplicates and mean values are shown. **(A)** Caspofungin resistance in RPMI/glucose medium with (left) and without (right) addition of bile. **(B)** Same as in **(A)**, but with amphotericin B. **(C)** Endpoint analysis (20 h) of caspofungin (5 μg/ml) resistance in presence of heat-inactivated (autoclaved) and native reconstituted bile in RPMI/glucose medium. **(D)** Endpoint analysis of caspofungin resistance (1 μg/ml) in presence of an ethylacetate extract made from crude bile. Bar diagrams in **(C)** and **(D)** represent mean + SD. Significance was calculated using the two-tailed Student’s *t*-test (^∗∗∗^*p* < 0.005, NS = not significant).

Resistance analyses were performed in broth dilution assays in which porcine bile (6% w/v) was tested for its protective effect against caspofungin (CAS) and amphotericin B (AMB). As shown in **Figures 1A,B**, *C. albicans* was sensitive against CAS at 1.25 μg/ml (MIC 0.125 mg/l), but nearly completely protected in the presence of bile even at 20 μg/ml. Similarly, *C. albicans* was sensitive at 0.25 μg/ml AMB (MIC = 0.063 mg/l), but protected in the presence of bile although protection against AMB was less pronounced compared to CAS.

Bile solids consist of about 60% bile salts, 12% fatty acids, 9% cholesterol, 7% proteins, and 3% phospholipids and bilirubin (Farina et al., 2009). To elucidate the contribution of bile proteins (Dietschy, 1968; Pasternak et al., 2014) in protection, bile was heat-inactivated by autoclaving. Autoclaved bile showed the same protection as non-treated bile, indicating that no heat-sensitive compounds were required for protection (**Figure 1C**). Furthermore, an ethyl acetate extract prepared from bile did not protect against CAS (**Figure 1D**). These analyses imply that neither proteins nor fatty acids, phospholipids or cholesterol are protective against antifungals.

### Conjugated Bile Salts Are Well Tolerated by *C. albicans*

**FIGURE 2:**
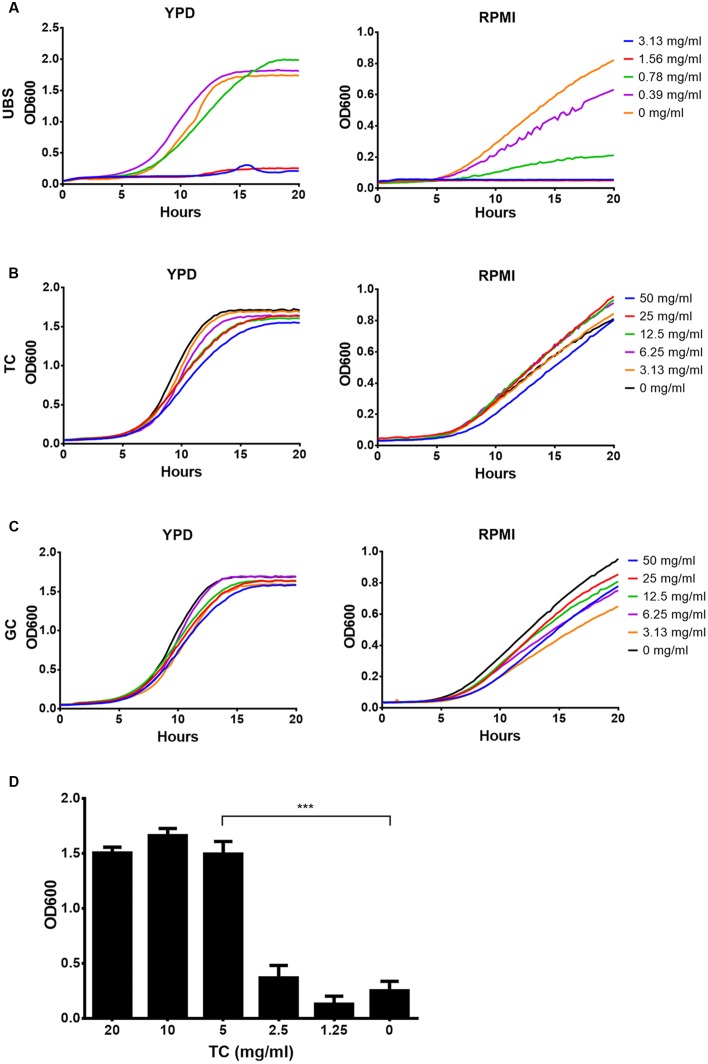
**Effect of unconjugated and conjugated bile salts on growth of *Candida albicans*.** Mean data from three independent experiments are shown. Assays were performed in YPD or RPMI medium. **(A)** Growth in the presence of a serial dilution of unconjugated bile salts (UBS) consisting of a 1:1 mixture of sodium cholate and sodium deoxycholate. **(B)** Growth in presence of sodium taurocholate (TC). **(C)** Growth in presence of sodium glycocholate (GC). **(D)** Yeast cells were grown in the presence of 2 mg/ml UBS and different concentrations of TC. Bar diagrams show the mean + SD. Significance was calculated by two-tailed Student’s *t*-test (^∗∗∗^*p* < 0.005).

Based on analyses above, we focused on conjugated and unconjugated bile salts. First, sensitivity analyses of *C. albicans* against possible toxic effects of unconjugated choline and deoxycholine in comparison to taurine and glycine conjugated choline were performed in YPD and RPMI medium (**Figure 2**). A mixture of choline and deoxycholine restricted growth in both media with an MIC of about 1.56 mg/ml (**Figure 2A**). In contrast, conjugated choline up to a concentration of at least 50 mg/ml was well tolerated, and no difference between taurocholate (TC) and glycocholate (GC) was observed (**Figures 2B,C**). Even more, 5 mg/ml of TC neutralized the toxic effects of 2 mg/ml of UBS (**Figure 2D**). Since conjugation of bile salts occurs directly within liver cells located at the periportal region of the liver conjugated bile salts are predominantly found in bile fluids (Solaas et al., 2000) with a concentration of about 40 mM (Begley et al., 2005). De-conjugation mainly occurs within the intestine by bacterial degradation (Begley et al., 2005). Therefore, although bile fluids within the intestine may contain a significant proportion of UBS, their toxic effects on *C. albicans* can be neutralized by the remaining conjugated bile salts.

### Conjugated Bile Salts Confer Resistance of *C. albicans* against Antifungals

**FIGURE 3:**
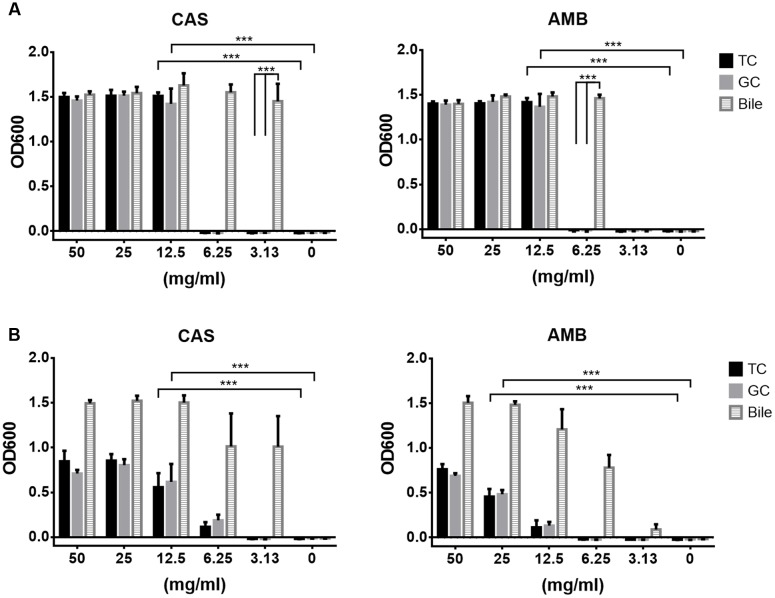
**Caspofungin and amphotericin B protection by bile, tauro- and glycocholate.** *C. albicans* yeast cells were cultivated in presence of a fixed concentration of caspofungin (CAS, 1 μg/ml) or amphotericin B (AMB, 0.5 μg/ml in RPMI and 2 μg/ml in YPD medium). Serial dilutions of either bile, taurocholate (TC) or glycocholate (GC) were added. Endpoint determinations after 20 h at 37°C are shown. **(A)** Analyses performed in YPD medium. **(B)** Analyses performed in RPMI/glucose medium. Bar diagrams show mean values + SD from three independent experiments. Statistical analyses were performed by using the two-tailed Student’s *t*-test (^∗∗∗^*p* < 0.005).

To determine the protective effect of conjugated bile salts in comparison to bile, sensitivity tests in YPD (**Figure 3A**) and RPMI/glucose medium (**Figure 3B**) against CAS and AMB were performed. Indeed both, TC and GC were able to protect from antifungals in a concentration-dependent manner. Bile exhibited a full protective effect at about 3.1–6.3 mg/ml against both drugs, whereas a slightly higher concentration of about 12.5 mg/ml of conjugated bile salts was required for protection.

### Protection Does Not Require Expression of Specific Resistance Associated Genes

**FIGURE 4:**
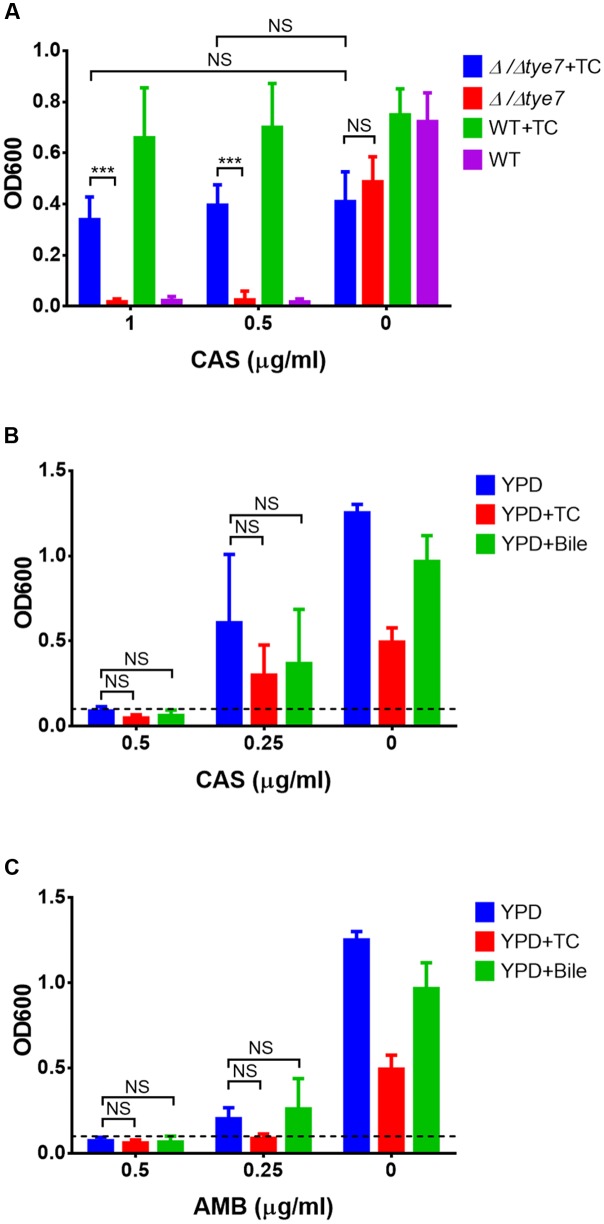
**Effect of *tye7* gene deletion and pre-adaptation to bile or taurocholic acid on protection against antifungals.** All data represent mean values + SD from three independent experiments and endpoint data after 20 h of incubation are shown. **(A)** Wild type (WT) and Δ/Δ*tye7* mutant were grown in RPMI/glucose medium with or without 25 mg/ml taurocholate (TC) and varying concentrations of caspofungin (0, 0.5, 1 μg/ml). **(B,C)** *C. albicans* yeast cells were pre-incubated in YPD medium with or without 25 mg/ml taurocholate (TC) or 12.5 mg/ml bile. For sensitivity testing wells were inoculated at an OD_600_ of 0.1 (dashed line) and varying concentrations of **(B)** caspofungin or **(C)** amphotericin B were added. Growth was assessed after 20 h of incubation. Statistical significance was calculated by using the two-tailed Student’s *t*-test (^∗∗∗^*p* < 0.005, NS = not significant).

Microarray analyses were performed to investigate the mechanism of antifungal protection in the presence of TC. Cells exponentially growing in YPD were shifted to RPMI/glucose medium with or without TC and gene expression was analyzed after 0.5 and 2 h of incubation. No clear pattern for the upregulation of genes typically involved in drug resistance was observed, and the drug efflux pumps and transporters, such as Cdr1, Cdr2, and Mdr1 (Nakamura et al., 2001; Watamoto et al., 2011) showed similar expression patterns in presence or absence of TC. However, expression of genes involved in sulfur amino acid metabolism were downregulated indicating that the sulfur content of taurine from TC was sensed (**Table 2**). In contrast, hyphae-associated genes, such as *ECE1* and *HWP1* as well as the global regulator Tye7 (Askew et al., 2009) were upregulated. To analyze the impact of Tye7 on antifungal protection a *tye7*Δ/Δ mutant of *C. albicans* (Homann et al., 2009) was tested for its resistance against caspofungin in the presence of TC. Although the *tye7* mutant showed general reduction in growth rate, TC-mediated resistance was comparable to that of the parental strain (**Figure 4A**). Thus, Tye7 is not important for protection.

**Table 2:** Expression of sulfur metabolism-related genes in *Candida albicans.*

Selected genes	TC (0.5 h)^1^	RPMI (0.5 h)	TC (2 h)^1^	RPMI (2 h)
*MET2*^a^	-2.31	-1.57	-2.52	-1.28
*MET3*^b^	-3.39	-1.86	-2.41	1.24
*MET10*^c^	-11.68	-5.22	-9.38	-1.93
*MET14*^d^	-2.06	-1.63	-2.43	-1.16
*MET15*^e^	-5.37	-3.11	-2.69	1.04
*MET16*^f^	-2.48	-1.86	-2.10	-1.80

*Expression levels are normalized against RNA from an exponentially growing YPD culture that was used as common reference.*

*^1^TC = 25 mg/ml.*

*^a^Homoserine acetyltransferase; ^b^ATP sulfurlyase of sulfate assimilation; ^c^sulfite reductase; ^d^putative adenylylsulfate kinase; ^e^O-acetylhomoserine O-acetylserine sulfhydrylase; ^f^putative 3’-phosphoadenylsulfate reductase.*

We subsequently tested if pre-incubation with bile or TC protects *C. albicans*. Cells were pre-grown in the presence of TC or bile, washed and challenged with CAS or AMB. Since both drugs remained inhibitory (**Figures 4B,C**), no pre-adaptation toward antifungals can be induced by bile or conjugated bile salts.

### Conjugated Bile Salts Block Quorum Sensing by Farnesol

**FIGURE 5:**
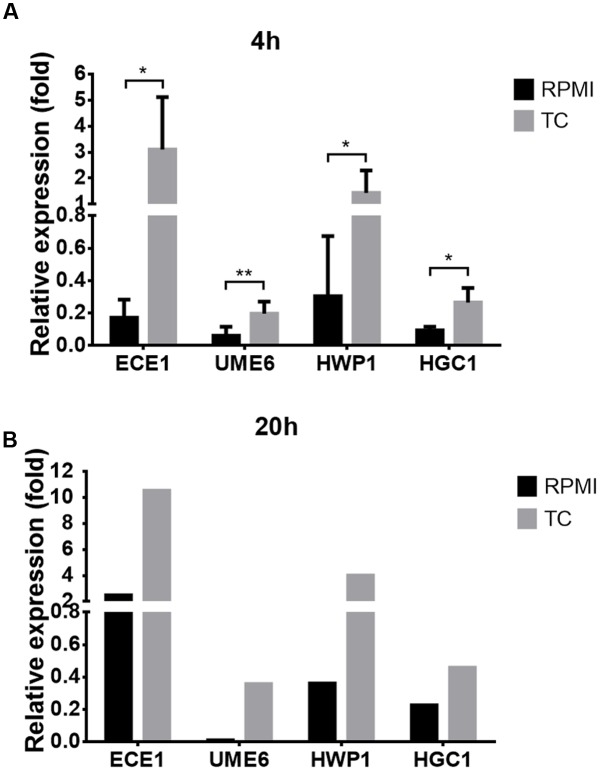
**Expression of hyphae-specific genes in the presence of conjugated bile salts.** Expression of hyphae-specific genes *ECE1*, *UME6*, *HWP1*, and *HCG1* was analyzed by qRT-PCR and normalized against actin gene (*ACT1*) expression. Cells were grown at 37°C in RPMI/glucose medium with or without the addition of 10 mg/ml taurocholate (TC). **(A)** Expression analysis at 4 h from three biological replicates in technical duplicates. Bar diagrams show mean values + SD. Statistical significance was calculated by using the two-tailed Student’s *t*-test with ^∗^*p* < 0.05 and ^∗∗^*p* < 0.01. **(B)** Expression analysis at 20 h from one biological sample as technical duplicate.

**FIGURE 6:**
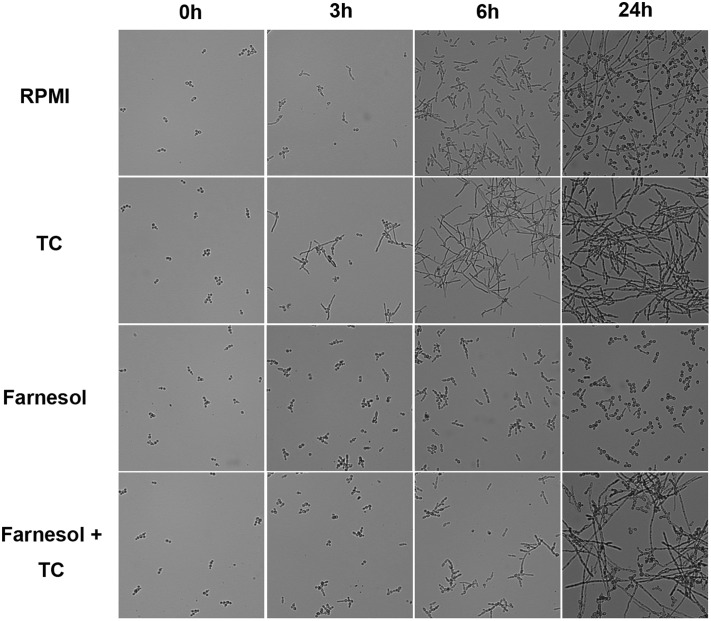
**Morphology of *C. albicans* in the presence of taurocholic acid and farnesol.** *C. albicans* yeast cells were incubated in RPMI medium at 37°C in the absence or presence of 10 mg/ml taurocholate, 200 μM farnesol or combinations thereof. Morphology was analyzed by light microscopy at the time of inoculation and at 3, 6, and 24 h.

Microarray experiments revealed a strong upregulation of hyphae-specific genes in presence of TC (**Table 3**). qRT-PCR analyses confirmed upregulation of hyphae-associated genes after the transfer to TC containing medium at 4 and 20 h (**Figure 5**). This high gene expression levels during prolonged incubation was unexpected since *C. albicans* produces the quorum sensing molecule farnesol that triggers the reversal to yeast cells when exceeding a certain threshold level (Ramage et al., 2002; Langford et al., 2009). Therefore, morphological analyses were performed in RMPI/glucose medium, which triggers the initial formation of hyphae. Control cells cultivated without either farnesol or TC showed the expected morphological transitions (**Figure 6**): Pseudo- and true hyphae formed at early time points with an accumulation of hyphae after 6 h. However, after 24 h a mixture of yeast and hyphal cells was detected, indicating a functional quorum sensing mechanism. Cells cultivated in the presence of TC formed hyphae at early time points that failed to switch back to yeast cells, and a 24 h culture consisted solely of hyphae (**Figure 6**). In contrast, cells cultivated in the presence of farnesol remained in yeast form throughout the 24 h observation period (**Figure 6**). In the presence of both, farnesol and TC the transition to hyphae was slightly delayed compared to TC alone. However, hyphae were produced and even after 24 h only small numbers of yeast cells were observed (**Figure 6**). This indicates that TC efficiently blocks farnesol signaling and implies that a similar mechanism might hold true for the protection from antifungals. However, it should be mentioned that TC by itself does not act as a stimulus for hyphae formation. When cells were cultivated in YPD medium no transition into the hyphal morphology was observed by the addition of TC (not shown). Therefore, experiments on drug sensitivity performed in YPD medium resemble planktonic yeast growth, whereas those in RPMI medium resemble hyphal growth within tissues.

**Table 3:** Expression of hyphae-associated genes in *C. albicans*.

Selected genes	TC (0.5 h)^1^	RPMI (0.5 h)	TC (2 h)^1^	RPMI (2 h)
*ECE1*^a^	14.56	1.39	27.49	3.14
*HWP1*^b^	1.15	-1.05	3.70	1.48
*HYR1*^c^	2.61	2.10	2.29	2.24
*SOD5*^d^	4.08	1.28	6.54	1.70
*IHD1*^e^	-1.55	-1.24	1.22	-1.25
*RBT1*^f^	1.28	1.15	1.53	1.26

*Expression levels are normalized against RNA from an exponentially growing YPD culture that was used as common reference.*

*^1^TC = 25 mg/ml.*

*^a^Hyphal-specific cell wall protein; ^b^hyphal cell wall protein; ^c^hyphal-induced GPI-anchored cell wall protein; ^d^copper- and zinc-containing superoxide dismutase (induced by hyphal growth); ^e^putative GPI-anchored protein (greater transcription in hyphal than yeast); ^f^cell wall protein with similarity to Hwp1p.*

### Bile and Conjugated Bile Salts Block Rhodamine 6G and Crystal Violet Absorption

**FIGURE 7:**
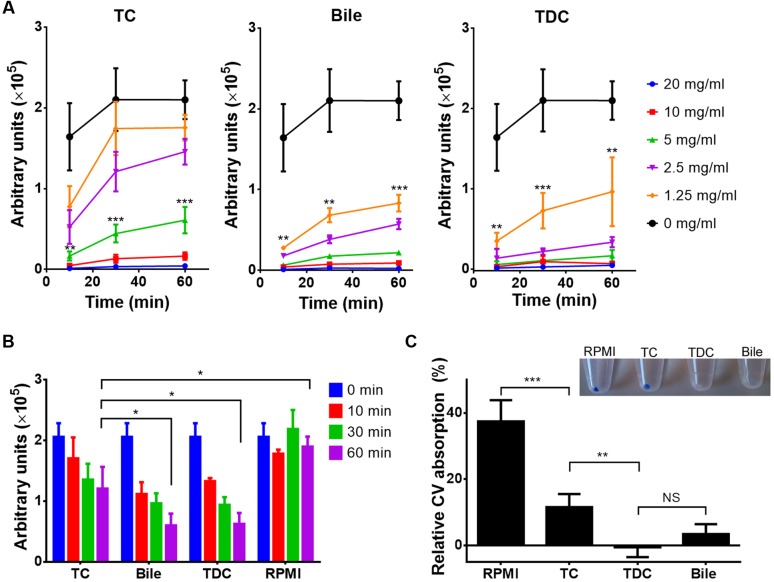
**Uptake and efflux of rhodamine 6G and crystal violet (CV) in the presence of bile or conjugated bile salts.** All data represent mean values + SD from three independent experiments. The Student’s *t*-test was used for calculation of statistical significance (^∗^*p* < 0.05, ^∗∗^*p* < 0.01, ^∗∗∗^*p* < 0.005, NS = not significant). **(A)** Time-dependent rhodamine 6G (R6G) uptake by *C. albicans* cells in the presence of serial dilutions of either taurocholate (TC), bile or taurodeoxycholate (TDC). Cells were harvested at indicated time points, washed and OD_600_-normalized R6G fluorescence was measured. Low fluorescence indicates low R6G absorption. Curves with asterisks indicate concentrations of TC (5 mg/ml), bile (1.25 mg/ml), and TDC (1.25 mg/ml) at which absorption significantly differs from the control. **(B)** R6G efflux assay. Yeast cells were pre-loaded with 1 μM R6G and shifted to R6G-containing media containing a fixed concentration (5 mg/ml) of either TC, bile or TDC. Efflux in presence of bile or TDC is significantly faster than with TC. **(C)** CV absorption by *C. albicans* in the presence or absence of 5 mg/ml of TC, TDC or bile. Inlay shows cell pellets after 30 min incubation with 2 μg/ml CV. The bar diagram shows the relative CV absorption from culture supernatants after 30 min of incubation in the presence of 4 μg/ml CV.

Rhodamine 6G (R6G) is a fluorescent dye that is used as a model compound in drug influx and efflux studies (Nakamura et al., 2001; Saengkhae et al., 2003). In- and efflux of R6G was investigated in the presence of bile, TC or taurine conjugated deoxycholic acid (TDC), another major bile component. As shown in **Figure 7A**, influx of R6G was blocked by all three additives, although a clear dependence on the specific bile compound was observed. While at least 5 mg/ml of TC were required to block the uptake of R6G, 1.25 mg/ml of bile or TDC already blocked influx. Similarly, all three compounds triggered the efflux of R6G when applied at a concentration of 5 mg/ml, with a significantly faster efflux in presence of bile or TDC than with TC (**Figure 7B**). These results suggested that bile and conjugated bile salts can protect *C. albicans* from antifungals in a dose-dependent manner by interacting with drugs in the growth medium. Results also implied that TDC might possess a higher protective potential than TC. Indeed, when antifungal protection against amphotericin B and caspofungin was tested with all three compounds, bile and TDC required the same concentration for protection (2.5 mg/ml for amphotericin B and 5 mg/ml for caspofungin), whereas higher concentrations were required for protection by TC (10 mg/ml). Finally, when the absorption of the toxic compound CV was investigated, TC, TDC and bile significantly reduced CV absorption, with TDC and bile being more effective than TC (**Figure 7C**).

### Critical Micelle Concentration Correlates with Antifungal Protection

**FIGURE 8:**
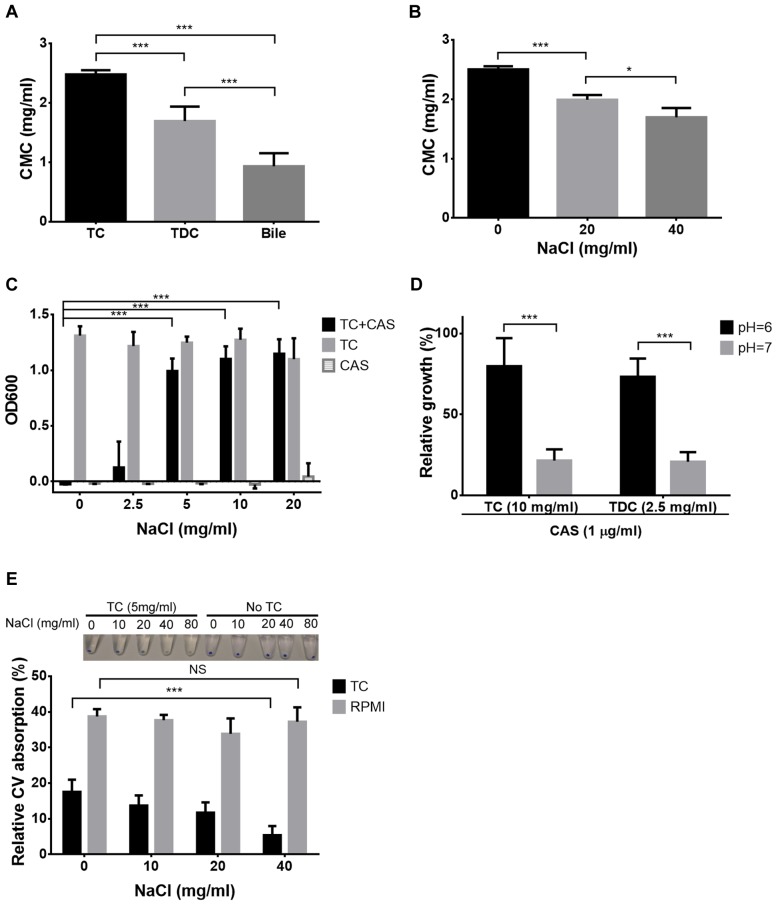
**Micelle formation and antifungal protection.** All diagrams show mean values + SD from three independent experiments. **(A)** Determination of the critical micellar concentration (CMC) of TC, TDC and bile by using an eosin Y assay. CMC values were calculated from trend lines based on the logarithm of the sample concentration against absorbance at 542 nm. **(B)** Effect of increasing ionic strength on the CMC of TC. **(C)** Effect of increasing salt concentration on TC-mediated protection against caspofungin (CAS). Media contain a fixed concentration of either 10 mg/ml TC, 2 μg/ml CAS or a combination of both. **(D)** Effect of pH on TC- (10 mg/ml) and TDC- (2.5 mg/ml) mediated protection against 1 μg/ml CAS. Data were normalized against control growth in the absence of CAS. **(E)** CV absorption in the presence of 5 mg/ml TC at different ionic strength. Inlay photo shows the NaCl-dependent decolourisation of cell pellets. The bar diagram visualizes the relative CV absorption from the culture medium after 30 min of incubation. Statistical analyses were performed by the two-tailed Student’s *t*-test (^∗^*p* < 0.05, ^∗∗∗^*p* < 0.005, NS = not significant).

Bile solubilises hydrophobic and amphiphilic molecules by forming mixed micelles (Begley et al., 2005). However, micelle formation only occurs when a so-called critical micellar concentration (CMC) is exceeded. Due to the different concentrations of bile, TC and TDC required in the R6G and CV in- and efflux experiments, CMC values were determined for all three compounds (**Figure 8A**). Indeed, CMC values of bile and TDC were significantly lower than that of TC with bile showing the lowest CMC. To confirm that CMC determines protection, conditions that reduce CMCs such as increased salt concentration or lowered pH were tested (Sugihara et al., 1982) (**Figure 8B**). Indeed, lower concentrations of TC or TDC were required for protection against CAS when either ionic strength was increased or the pH lowered (**Figures 8C,D**). Similarly, absorption of CV in presence of 5 mg/ml TC was reduced when the salt concentration was increased (**Figure 8E**). This confirms trapping of antifungals in micelles, thereby reducing their bioavailability.

### Small and Hydrophilic Drugs Remain Active in Presence of Conjugated Bile Salts

**FIGURE 9:**
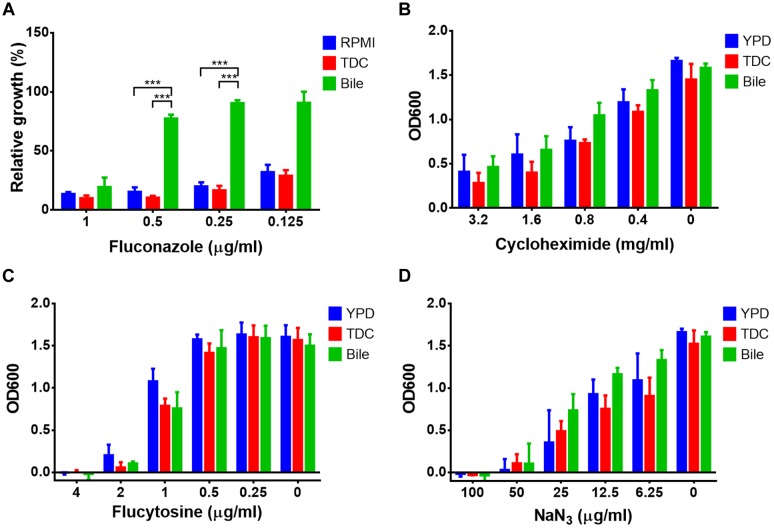
**Bile- and conjugated bile salt-mediated resistance against small and hydrophilic antifungals.** Endpoint determination of growth after 20 h incubation at 37°C. Bile (12.5 mg/ml) and taurodeoxycholic acid (TDC, 12.5 mg/ml) or media controls were treated with serially diluted **(A)** fluconazole, **(B)** cycloheximide, **(C)** flucytosine, or **(D)** sodium azide. Bar diagrams represent the mean values + SD from three independent experiments. The Student’s *t*-test was used for calculation of statistical significance (^∗∗∗^*p* < 0.005).

As bile and conjugated bile salts are very effective in solubilising hydrophobic and amphiphilic compounds, we assumed that small and hydrophilic compounds might be less efficiently trapped in micelles. Therefore, the protective effect against other antifungal compounds, such as fluconazole, cycloheximide, flucytosine, and sodium azide was tested. The efficacy of fluconazole was dramatically decreased in the presence 12.5 mg/ml bile, whereas the same concentration of TDC was not protective (**Figure 9A**). When cycloheximide (**Figure 9B**), flucytosine (**Figure 9C**), or sodium azide (**Figure 9D**) were tested, neither bile nor TDC were protective. This suggests that bile and conjugated bile salts cannot efficiently trap small and hydrophilic compounds in mixed micelles.

## Discussion

Recently, studies on a murine infection model showed that *C. albicans* is protected from antifungals inside the gall bladder and shed cells survive the passage through the bile duct and intestine (Jacobsen et al., 2014). Therefore, gall bladder colonization in patients might act as a reservoir for re-colonization of the intestine after discontinuation of antifungal therapy. Here, we show the relevance of micelle formation by conjugated bile salts as the major mechanism of protection.

Bile salts have the potential to inactivate pathogens (Hofmann, 1999) and, in agreement, UBS showed toxic effects on *C. albicans*. However, conjugation of bile salts with either taurine or glycine reduced their toxicity and, in a mixture, were able to detoxify UBS. Therefore, conjugation of bile salts seems essential for *C. albicans* to colonize the gall bladder. However, a specific transcriptional response toward conjugated bile salts seems of minor importance for the protection from antifungals. Despite upregulation of glycolysis and hyphae-associated genes no clear pattern for the upregulation of resistance-associated genes was observed (Mathe and Van Dijck, 2013). Some of the observed changes in gene expression may result from blocking of the quorum sensing molecule farnesol (Wongsuk et al., 2016), which is in agreement with the early formation of true hyphae and cultures remaining in hyphal morphology even at high cell densities. This effect of conjugated bile salts could possibly counteract the known synergistic effects of farnesol with antifungals in the treatment of fungal (Katragkou et al., 2015) or mixed species (Fernandes et al., 2016) biofilms.

In our experiments bile or conjugated bile salts prevented influx and promoted efflux of drugs as shown for the model compounds R6G and CV. While bile salts can act as so-called “enabling formulations” to increase the bioavailability of drugs with poor dissolution characteristics (Buckley et al., 2013), bioavailability requires the release of drugs from micelles for crossing membranes of target cells. This is dependent on the equilibrium of drugs free in solution and drugs bound in micelles (Yano et al., 2010). However, significant differences in the encapsulation capability between bile and its conjugated bile salts seem to exist. When testing 30 different compounds, free drug concentration of some compounds was reduced by either bile or a mixture of taurocholate and lecithin, whereas other drugs were only encapsulated by bile (Berginc et al., 2012). This reflects our investigations, in which CAS and AMB were encapsulated by both, bile and conjugated bile salts, whereas fluconazole remained active with TC or TDC, but was inactivated by bile. This may be a result of the more complex composition of bile, which forms more variable mixed micelles that possess higher drug binding capacities than those made of pure conjugated bile salts. The presence of cholesterol in bile samples may also at least partially contribute to the increased protective effect of bile against fluconazole. Investigations on *Aspergillus fumigatus* showed that cholesterol increased resistance against itraconazole by incorporation of cholesterol into the fungal cell membrane. However, a much lower protective effect was observed when voriconazole was used, which indicates that cholesterol incorporation on its own is not sufficient for a general azole resistant phenotype (Xiong et al., 2005). In this respect, it should be noted that the ethyl acetate extract of bile containing phospholipids, fatty acids and cholesterol was not protective against various drugs tested. Nevertheless, future studies will have to investigate the increased efficiency of drug inactivation from mixed micelles by combining conjugated bile salts with phospholipids, unsaturated fatty acids and cholesterol.

Caspofungin, which shows excellent efficacies in treatment of systemic infections gets inactivated by bile and bile salts and, in agreement, failed in a case series for treatment of biliary tract infections (Domagk et al., 2006). In addition, a recent study on fungal cholangitis patients after liver transplantation revealed a reduced bioavailability of different AMB formulations in bile. The maximum concentration of AMB detected in one of the human bile samples was 1.28 mg/l, but *in vitro* studies showed that up to 5 mg/l of AMB added to human bile samples did not inactivate *C. albicans* or other *Candida* species leading to the conclusion that a reliable treatment success on fungal cholangitis with AMB cannot be anticipated (Welte et al., 2015). This underlines our results on the reduced bioavailability of antifungals by micelle formation. Importantly, a recent study on echinocandin resistant *Candida spp.* in liver transplant recipients revealed the emergence of resistant isolates within one months after initiation of treatment (Prigent et al., 2016). At least three resistant *C. albicans* isolates were isogenic to susceptible isolates from the same patient, indicating that resistance occurred under the selective pressure of echinocandin treatment. While some resistant *Candia spp.* were isolated from multiple body site, resistant isolates were overrepresented in samples collected from the anus region. It was speculated that sub-inhibitory drug concentrations may be present in the digestive system promoting the emergence of resistance (Prigent et al., 2016). In our previous study (Jacobsen et al., 2014), we found that *C. albicans* cells persisting under antifungal therapy in the gall-bladder are shed by bile and survive the intestinal passage. This shedding possibly leads to a re-colonization of the intestine. In agreement with the speculation above, encapsulation of drugs in micelles lowers their bioavailability to a sub-inhibitory concentration that might indeed facilitate the emergence of resistant isolates either directly within the biliary system or in the intestine. If this holds true, efficient monitoring of fungal bile infections in patients at risk for invasive fungal infections should be considered.

The strong reduction of antifungal efficacy of several drugs in the presence of bile may make it difficult to resolve fungal infections within the biliary tract. However, neither bile nor conjugated bile salts were able to efficiently encapsulate small and hydrophilic compounds, such as cycloheximide, flucytosine, or sodium azide. Therefore, if susceptibility profiling allows, antifungals that are difficult to trap in micelles should be considered when treating fungal infections of the biliary tract.

## Author Contributions

S-HH designed and performed experiments and evaluated data, SB planned and evaluated microarray experiments, MB designed experiments, evaluated data, and wrote the manuscript. All authors critically revised the manuscript, approved it for publication and agree to be accountable for the content of the work.

## Conflict of Interest Statement

The authors declare that the research was conducted in the absence of any commercial or financial relationships that could be construed as a potential conflict of interest.

## Footnotes

1 www.ebi.ac.uk/arrayexpress
